# Living with epidermolysis bullosa: Daily challenges and health‐care needs

**DOI:** 10.1111/hex.13006

**Published:** 2019-12-23

**Authors:** Sandra Kearney, Ann Donohoe, Eilish McAuliffe

**Affiliations:** ^1^ IRIS Centre School of Nursing, Midwifery and Health Systems University College Dublin Dublin Ireland; ^2^ School of Nursing and Midwifery Royal College of Surgeons in Ireland Dublin Ireland

**Keywords:** barriers to care, Epidermolysis bullosa, health‐care needs, lived experience

## Abstract

**Background & Objective:**

Epidermolysis bullosa (EB) is the term used for a group of genetic skin fragility disorders. For those living with EB, pain represents a constant challenge, with blistering and tasks such as changing dressings, adding to the distress. This paper focuses on describing and exploring the health‐care needs of children, adults and families who are affected by EB. The specific aim of the paper is to identify the needs of the EB population with a view towards informing the development of a community liaison service to support adults living with EB and the parents/carers of children living with EB.

**Setting and Participants:**

Interviews with six adults and the parents of eight children with EB were conducted. The data were analysed thematically. All participants were resident on the island of Ireland and are therefore reflecting on services in this geographic region.

**Results:**

Participants’ needs were grouped into five themes: support managing physical health‐care issues; access to community/home‐based services; EB‐specific information and psychosocial support; effective interaction with health‐care professionals; and advice regarding benefits and entitlements.

**Discussion and Conclusions:**

This article represents the health‐care needs and preferences of a broad spectrum of those with EB, highlighting the need for a comprehensive service regardless of the severity of the condition.

## 1 INTRODUCTION

Epidermolysis bullosa (EB) is the term used to describe a group of rare genetic skin disorders which result in an extreme susceptibility to blister. There are more than 30 subtypes identified, and these are generally categorized into four groups: EB simplex (EBS); dystrophic EB (DEB); junctional EB (JEB); and Kindler syndrome.^1^ EBS is considered the mildest form of EB, with blistering focused mainly in the feet and hands. For those with more severe subtypes, pain is a central part of the condition and individuals require daily skin care routines and bandaging on most parts of the body.^2^, ^3^ Treatments focus on prevention of blisters, use of appropriate bandages and ointments and support for physical and mental well‐being,^4^ requiring the services of a wide range of disciplines, such as dermatology, nursing, dietetics, occupational therapy and physiotherapy. Furthermore, holistic approaches to care such as psychological and social support are also critical.^5^ Co‐ordinated care between specialized and community‐based services is necessary to enable the seamless provision of care.^5^ In Ireland, specialist care is delivered by two multidisciplinary teams, one for children and one for adults. The patient representative organization DEBRA also offers advocacy and community outreach services for individuals with EB.

Despite the obvious need, research into the challenges faced by this population in relation to their health‐care provision has been limited. An analysis of EB health‐care support conducted in Ireland suggested access to specialist EB support staff in the hospital, and the community was important.^6^ This study also emphasized challenges in relation to accessing services, as well as the emotional and social cost to caregivers.^6^

A qualitative examination of the problems faced by children with EB in the Netherlands found that difficulties dealing with itchy skin, being in pain and others not understanding EB were particularly challenging. In this study, eleven children with various types of EB were interviewed using a semi‐structured guide. Results also indicated that participants felt that others thought EB was contagious, hindering normal social interactions.^7^ Similarly, the experiences of young people aged 10 to 14 years old with EB in the UK were explored qualitatively. Participants reported that they felt different, were treated negatively by peers and found it difficult to manage others not understanding EB.^8^ The findings from these studies highlight the importance of social and psychological support for EB.

Another study examined the concerns of parents of children with EB in the Netherlands. In total, eleven families were interviewed, with the main difficulties experienced relating to their child being different from others and the pain their child experienced.^9^ This research also documented family difficulties, as well as restrictions on leisure time and employment. Participants also highlighted difficulties in relation to care provision, particularly when interacting with unknowledgeable care providers.^9^ This study identified the need for a care manager who could answer questions that families have, provide support and signpost to other services.

A survey study from the United States found high levels of marital breakdown in families with children with EB.^10^ In addition, the authors also found that a significant number of those with children with more clinically severe types of EB chose not to have more children. The authors concluded that more support needed to be available for these parents.

A qualitative interview study by Dures et al^11^ examined the psychosocial impact of EB on 24 adults (14 women and 10 men) with EBS, DEB and JEB. This study found that adults existed on a continuum from those who felt they could effectively manage the physical aspects of their condition to those who felt that EB dominated their life. The authors also found that EB had a disabling impact on adults physically and socially, with the visual difference of EB adversely impacting normal social interactions. Suggested support needs ranged from a preference for skill‐based interventions and peer support to psychological support.

It is evident from the literature reviewed that the health‐care needs of people with EB are under researched. In addition, most of the research conducted has focused on examining the impact of EB and its effects on daily living. Consequently, research that explores what individuals with EB want or need from services is limited. This study also offers those affected by EB a platform to articulate their health‐care needs. Furthermore, this study demonstrates how participatory action research can be used to develop an effective partnership between key stakeholders to affect change within a health‐care system. Finally, it is proposed that the findings in this paper could provide health professionals with an insight into the lives of people with EB, so that effective services can be provided to meet the needs of this population in the home, in the community and in the hospital setting.

## 2 METHOD

The research was designed in line with the principles of Participatory Action Research (PAR) which focuses on making action‐based change with community stakeholders.^12^ The primary purpose of PAR is to guide social change, with recommendations that lead to direct action. The overall study sought to provide evidence to increase resources for an EB Outreach service,^13^ and as such, it was felt that PAR was optimal for this purpose. A key strength of PAR is that it brought all stakeholders together within a steering committee which included representation from two EB multidisciplinary teams, patient representatives, the patient organization DEBRA and the research team.

The research team felt that the most effective way to answer the overall research question was through interviews with adults with EB and parents of children with EB, and the steering committee concurred with this decision. The study therefore has an inductive, exploratory design. Serious consideration was also given to a variety of other methods, but these were ruled out for a variety of reasons. For example, the EB population in Ireland is relatively small. Hence, a quantitative study would have had limited validity and would have lacked the richness of data generated by a qualitative approach. In addition, as families and individuals with EB are time poor and geographically dispersed, it would also have been logistically difficult to run focus groups.

The steering committee also gave serious consideration to the inclusion of children in the study. However, there were legitimate concerns raised regarding the challenges, both ethical and logistic, in including children in this type of study given the onerous care schedules of children with EB. Further concerns were raised as to the legitimacy of such an approach given that it was anticipated that the service would be accessed primarily by parents and adults.

Consequently, it was decided that adults and parents would be best placed to participate in the interview process. Although children's voices would add substantially to our knowledge and understanding of the challenges of living with EB, the purpose of this research was to inform the development of a community liaison service to support adults living with EB and the parents/carers of children living with EB.

### 2.1 Ethics

Ethical approval for the study was obtained from three research ethics committees including the participating university and two hospitals. Participants gave informed written and verbal consent prior to participating in the interview.

### 2.2 Recruitment

Information packs were sent on behalf of the university to potential participants registered with EB centres in Ireland. This included adults with EB (46 Packs) and the parents of children with EB (48 Packs). This pack outlined the purpose of the study and asked individuals to contact a named researcher if interested in taking part. After this first contact, individuals were given time to decide if they still wished to participate. All individuals who contacted the researchers participated. The sample was therefore a convenience sample.

### 2.3 Data collection

Semi‐structured interviews commenced started in November 2017 and were completed by May 2018 when no further expressions of interest were received. In total, eight mothers and two fathers of children with EB participated, with children ranging in age from three to seventeen. Six adults with EB also participated in the study (three males and three females) and were aged between twenty‐two and sixty‐seven. Interviews were audio‐recorded and subsequently transcribed. The interview guide was adapted from previous work on service needs for those with chronic conditions.^14^ The interview guide was adapted from a previous study which focused on exploring continuity of care for children with complex chronic health conditions. Demographic information was also collected.

### 2.4 Data analysis

A six‐step thematic analysis approach was used to identify and analyse patterns in the data and to construct a rich narrative account.^15^ Thematic analysis is used when little is known about the subject. This approach enables the construction of knowledge to be guided by the participants’ data rather than any pre‐determined constructs (as might be the case in framework or other forms of top down analysis). As the aim of the study was to allow the views of those living with EB to inform the design of a new EB service, it was important to ensure that any pre‐determined ideas about such a service would not influence the findings. Thematic analysis was selected as an approach to minimize this risk. Interview transcripts were openly coded using an inductive approach, guided by the overall research question. The first and second authors conducted the initial coding and generated preliminary themes, while the third author sense checked the themes to ensure a coherent narrative. In the initial stages, the transcripts were read repeatedly to gain an in‐depth knowledge of the data, with the data then coded by utilizing the software Nvivo. Codes with a similar content that were prevalent throughout the transcripts were combined into preliminary themes, particularly those relating to care needs. Potential themes were reviewed against the coded data and the dataset overall to make sure they reflected the transcripts. These themes were then reviewed and combined resulting in the generation of five overall themes. Further refinements were made so that the content of the theme, direct quotations and the theme names were consistent.

## 3 RESULTS

The needs of individuals with EB were grouped into five themes identified by researchers: (a) support managing physical health‐care issues; (b) access to community/home‐based services; (c) EB‐specific information and psychosocial support; (d) effective interaction with health‐care professionals; and (e) advice regarding benefits and entitlements.

### 3.1 Theme 1: Support managing physical health‐care issues

Both adults and parents highlighted the need for appropriate support with physical care needs. However, differing levels of need existed depending on the severity of EB and the cohort. Those with more clinically severe EB required regular, continuous care, while those with EB simplex in both cohorts required more sporadic care, particularly with infected wounds. Adults also tended to manage the condition themselves, with parents having more consistent contact with care providers.

Bandage changes were a particularly difficult task for parents of children with clinically more severe types of EB and involved intense physical pain for the child but were necessary to avoid serious infections. One participant spoke of the suffering their child experienced during bandage changes, and on the occasions when supported by a nurse, it was sometimes possible to leave the room when this suffering became too much to bear. This participant also discussed how when supported by an EB nurse she ‘looked forward to the day she was coming’ because ‘it took the burden off me’ (Parent Participant 2). Parents discussed in detail how EB had consumed their life and discussed the daily struggle they faced. They thought of little else except EB and had no free time for doing normal everyday activities:

We do nothing outside of EB because we don’t have time. Our day is just completely consumed by bandaging or soaking… (Parent Participant 1)

For many adults and parents of children with EB simplex, EB was described as a condition that needed monitoring and active management. Generally, adults felt that they could manage EB independently, without the need for outside support. However, several participants highlighted one of the occasions where they would have appreciated support during serious infections or non–EB‐related hospital admissions.

It just would be one of those circumstances that is dire, but it would be brilliant [for someone] to say don’t worry […] you don’t have to go out and get more fatigued.(Adult Participant 3)

Most adults carefully monitored their condition to avoid these instances; however, when they did occur, they resulted in extreme pain, missed work or college and would sometimes necessitate contacting unspecialized care providers, which most adults sought to avoid due to the difficulty explaining the seriousness of what would appear to treating health‐care professionals (HCP), as a minor skin infection:

So even if you went into A&E, they do not know what to do with you. I did that once, that did not work at all and my feet were really badly blistered (Adult Participant 2)

### 3.2 Theme 2: Access to community/home‐based services

Participants discussed their desire for services that were accessible, in their community setting or within their home. Individuals reported on the difficulties faced when accessing EB services based within the hospital and/or community settings.

The need for more locally based care was highlighted by a family who were required to travel substantial distances to hospital and who felt that this journey adversely affected their child's wound care. This geographical isolation from specialized services left some feeling that home visits by an EB professional would substantially benefit care:

It would’ve been in the early stages, even just for one or two visits and just from an education point of view. I would’ve loved someone there to tell me about the different dressings… (Parent Participant 6)

One participant also noted that her child spent substantial amounts of time attending hospital appointments but felt that this setting may not be an appropriate place to discuss the emotional aspects of EB.

Going to [avail of psychological services] in a hospital setting, to me is not ideal. I’d like to see it outside in a much more gentle space, a good space… (Parent Participant 2)

Another parent also voiced concern about attending appointments in a local hospital, as she feared her child catching a cold and how, if this did happen, it would have a detrimental impact on wound management.

I just don’t like bringing her in there if she’s unwell, I don’t want her getting this awful vomiting bug that’s around.(Parent Participant 1)

Private services were utilized when difficulties arose with accessing appropriate publicly funded services. One parent accessed occupational therapy and other holistic support privately due to lack of public services. Another adult commented that she preferred using specialized private chiropody services because she could easily park beside it, limiting the distance she spent walking. This contrasted with accessibility issues with the main hospital setting which required walking a long distance from a car park to a clinic which was ‘not very helpful for people with sore feet’ (Adult Participant 2).

### 3.3 Theme 3: EB‐specific information and psychosocial support

Participants in both cohorts placed emphasis on their need for up‐to‐date information particularly in relation to physical care needs. Participants also highlighted the social and emotional impact of EB with some expressing the need for improved service provision.

Both cohorts had a strong desire for practical information which helped with everyday aspects of living with EB, particularly appropriate clothes and shoes which could minimize blistering. However, others also discussed the need for information around managing wound care, serious infections and applying bandages, with many feeling that they lacked these skills and required sessions focusing on these issues:

How to burst a blister, how to dress it, things to look out for, how to look after wounds and how to look after skin (Parent Participant 7)

Both cohorts discussed infections which they felt unable to deal with and feared the consequences of falling seriously ill. Most felt that they had limited options available to them with some reporting that they attended A&E during bad outbreaks. Parents had considerable knowledge in relation to their child's wounds but sometimes feared the worst during the long process to confirm infections:

It’s always five days and by that time if it was something serious, the child could be dead (Parent Participant 1)

Participants also reported the strain that having EB placed on family life and intimate relationships. Difficulties were particularly evident for those with clinically more severe forms of EB. Parents described time‐consuming dressing changes multiple times every week with caring responsibilities falling predominantly to the mother. This often resulted in feelings of isolation within the home with many having little free time for normal family life.

It’s every single aspect of every single minute of every hour, of every day, and unless you are living it […], you just would not have a clue (Parent Participant 2)

Participants in both cohorts felt it was impossible for other people to understand EB unless they had it or lived with it. Some talked of the difficulties this caused within personal relationships with one participant discussing how walking to the bus stop left her in intense physical pain, but her partner could not understand why this happened and offered unhelpful solutions.

Can you not walk slower? Can you not wear different shoes? (Adult Participant 5)

These types of responses often left participants upset and sometimes isolated within personal and wider family relationships and requiring emotional and social support. Some participants highlighted a desire for contact with other people with EB to lessen feelings of isolation and the lack of understanding associated with the condition.

The need for emotional support with the genetic nature of the condition was also highlighted. Participants discussed the guilt experienced when realizing they had passed EB onto their child. One parent felt that her family was left to decide whether to have more children despite the genetic implications of this decision.

There is no emotional support, there’s nothing at all for the carer (Parent Participant 2)

Parents talked about the informal support they received from friends and family. This included child‐minding, cleaning and help with bandaging. Despite this care, some parents were reluctant to leave their child with family members due to the guilt family members felt if a child got hurt. For those with children with more clinically severe forms of the condition, this meant that parents could never have family or friends babysit their child and left them feeling like they never got a break from their caring duties.

### 3.4 Theme 4: Effective Interaction with Health‐care Professionals

All participants availed of hospital‐based services, with most attending an annual EB multidisciplinary clinic. Participants were generally satisfied with these interactions and were kept updated in relation to new developments in EB treatments.

The hospital is my sanctuary, it’s where I receive my knowledge (Parent Participant 2)

The contribution of community‐based services such as schools, pharmacists and private chiropody services in addition to home‐based nurses and personal assistants was highlighted by participants. The positive contribution of HCPs in community settings who initially had no knowledge of EB was also discussed. These individuals made substantial efforts to understand the family's support needs, and when these HCPs were absent, parents were reluctant to engage with someone else.

None of them [other GPs in the practice] understand the condition at all except for her, she was the only one that understood anything about it but the rest of them were, oh yeah that’s fine but the next time you see them, they haven’t a clue what you were even saying (Parent Participant 6)

Parents also talked about HCPs who contributed to their child's care in ways that circumvented bureaucratic challenges, for example public health nurses taking extra care to ensure that essential supplies of bandages did not run out and specific ‘pharmacists who were always very good’ in supporting families with acquiring bandaging (Parent Participant 4).

This also extended to help and advice with wounds. One participant highlighted how a public health nurse came to understand the challenges associated with EB and visited every week during bandaging to give advice and to learn more about the condition.

In contrast however, participants discussed inconsistent care delivered in hospital and community‐based settings. Participants routinely encountered HCPs who had little or no knowledge of EB. Consequently, participants reported that they learned to only contact EB‐specific services. However, on occasion this was not possible, resulting in unproductive interactions with non‐specialist HCPs.

Participants also felt that their personal knowledge and expertise was ignored during health‐care interactions. This was sometimes accompanied by questions which indicated that HCPs felt that individuals were being overly protective about either their own skin or their child's skin. This was frightening especially within hospital settings where requests were sometimes not treated seriously. This feeling of frustration was compounded by that fact that both cohorts felt that they had to explain EB to every HCP they encountered. These experiences left participants hesitant to access services in the future due to the fear of being hurt within a hospital setting.

‘When they put those little things on to do ECGs, they tend to want to pull them off and I have to explain don’t pull them off because […] it actually does take the skin off with them’ (Adult Participant 3)

### 3.5 Theme 5: Advice regarding benefits and entitlements

One of the biggest challenges faced by participants was the bureaucracy surrounding the accessing of services. However, these challenges were not evenly distributed across both cohorts. The parent cohort more so than the adult participants felt let down by the administrative wing of the health service, particularly those with more clinically severe forms of EB.

Parents outlined intense bureaucratic battles to ensure they were supported by home‐based personal assistants and/or nurses. One parent felt that there was an information gap between leaving the hospital and bringing the child home, with little guidance on accessing services. The feeling that no one took responsibility within the health‐care system was a source of frustration on top of substantial caring duties:

The lack of support, fighting every, month in month out […], it was just such an emotional time, […] I wasn’t going to get through it if I didn’t get some type of support.(Parent Participant 2)

Parents also highlighted difficulties interacting with administrative staff who sometimes displayed little concern for individual care needs and instead focused on the expense associated with specialist medical supplies and did not understand:

How important these dressings were, that it’s the child’s second skin and that you cannot recycle them (Parent Participant 2)

Some parents talked about the paperwork they were given when their child was born. This administrative burden was continually added to as they were required to provide the same information repeatedly causing additional stress at a time when parents were coming to terms with the diagnosis that their child had received.

However, one issue that both cohorts shared was the difficulty in attempting to access free medical care through the means tested medical card. An adult participant reported that when he was younger, his family applied for a medical card for him, but he was rejected resulting in his family having ‘a hefty bill every week’ (Adult Participant 4). Participants felt this process was frustrating particularly for a chronic condition and felt that they should not have to fight for this:

We’ll just pay for it and forget it but then it means that the next kid that comes up behind you […] still has to fight the fight (Parent Participant 4)

## 4 DISCUSSION

The aim of this article was to identify the specific health‐care needs within the EB population with a view towards developing appropriate and accessible health services for EB patients and their families. Participants articulated care needs in five areas relating to support with physical care; access to community services; EB‐specific information and psychosocial support; appropriate interactions with professionals; and advice regarding benefits and entitlements. Previous research in this area has tended to focus on the difficulties associated with the condition.^7^, ^8^ However, individuals with EB have rarely been asked about their care needs. This article addresses that gap and highlights the diversity of need and shows the requirement for a comprehensive service for this population.

Participants in both cohorts articulated the difficulties they experienced in relation to physical care needs particularly when managing infected wounds. Parents of those with clinically more severe forms of EB struggled significantly with the pain their child experienced during bandage changes and appreciated the support they received. However, this support was often time‐limited with parents discussing their daily struggle. A study of parents’ experiences of having a child with EB had similar findings with many experiencing difficulties related to the pain their child experienced.^9^ This research also documented restrictions on leisure time and employment, which was evident within the current study.

In comparison, the adult cohort felt that they could successfully manage EB. This could possibly be attributed to the fact that most adult participants had EBS. Despite this, adults described one of the situations, predominantly with serious infections where they felt they had limited options in terms of immediate support. Brun et al^16^ found that pain can be significant enough during blister flare ups for those with EBS to cause individuals to miss education or work.

This study illustrates that patients deal with complex health‐care issues at home. One solution to this could be the development of a co‐ordinator role that bridges the gap between community and specialist services similar to a role that exists in EB services in the Netherlands.^17^ This role, which is advocated more widely internationally for patients with other rare conditions, includes co‐ordinating and communicating care needs to relevant care providers and acting as a point of contact.^18^

Participants expressed a preference for home‐based services and spoke of the challenges associated with accessing community‐based care, with most wishing to limit visits to hospital‐based settings. Difficulties associated with accessing allied HCPs within community‐based services in Ireland are a system‐wide issue, with further resources required to fully meet the needs of all patients within their own communities.^19^

The need for home nursing assistance was expressed where parents were involved in wound care, with previous research providing support for this resource. A Cochrane review examining home‐based nursing services for children with chronic illnesses indicated a reduced length of hospitalization following admission.^20^ Furthermore, a home nursing programme for patients with EB in Australia highlighted improvements in quality of life, better provision of support and enhanced family life management.^21^

Participants within the present study had high levels of information and psychosocial support needs, particularly in relation to bandaging and wound care. Feelings of isolation were also common with many participants identifying the need for more emotional and social support. Dures et al^11^ examined the psychosocial impact of EB on adults and highlighted a range of potentially beneficial supports such as counselling, social skills and assertiveness training. Furthermore, access to online peer support could allow individuals to share information about how they cope and perhaps feel less alone with their condition. Indeed, a recent study highlighted that online support can facilitate a greater understanding of rare conditions among parents of children with rare conditions.^22^

The present study also highlighted the psychosocial difficulties of caring for a child with EB. Previous research in this area has found that parents experience significant caregiver stress^9^ and this can have a negative impact on family functioning and the marital relationship.^10^ A recent survey conducted in Australia and New Zealand found that 46% of parents of children with rare conditions also reported feeling isolated and lonely.^23^ In order to alleviate the negative impact on family functioning and to support the informational needs highlighted, families should have timely access to patient advocacy groups, genetic counselling or social work services.

Participants in general were satisfied with the care they received from specialist services. However, participants reported less favourable interactions with non‐specialist practitioners. Previous research has identified similar issues, with Dures et al^11^ finding that adults often felt disempowered by HCPs when their own knowledge of EB was disregarded. van Scheppingen et al^9^ also reported parents of children with EB struggled with health‐care environments where there was limited knowledge of EB. It is important to note however that participants recognized that some community HCPs had purposively developed their knowledge and skills in relation to EB. This approach was deeply appreciated by members of both cohorts as it improved the delivery of care and subsequently facilitated the development of positive relations with these professionals.

A potential solution to this issue could be closer links between specialists and non‐specialists with previous research highlighting that specialist EB HCPs identified liaising with non‐specialists HCPs as a key aspect of their role.^24^ Furthermore, The National Rare Disease Plan for Ireland^25^ recognizes the need to integrate modules and clinical/practical experience relating in the curricula of relevant health‐related education/training programmes.

Participants had very little support accessing benefits and entitlements with some outlining stressful administrative processes. Individuals with EB could benefit from support navigating the health system. This could be achieved through referral to the patient organization DEBRA Ireland who have a dedicated family support service.

Numerous issues relating to the application and renewal of medical cards were discussed, frustrating participants who felt this process was unnecessary due to the chronicity of EB. Gowran et al^6^ highlighted the difficulties experienced by those without medical cards, arguing that free access should be offered to all with EB, regardless of means. As research has highlighted high costs in EB management, access to free medical care would substantially reduce this burden for individuals and families.^2^ Difficulty accessing medical cards for those with rare and chronic conditions is a wider issue within the health‐care system that needs to be resolved to improve the overall equity and quality of life for all rare disease patients in Ireland.

### 4.1 Limitations & Future Research

A limitation of the current study is that children were not included, nor were they given the choice to participate. As the current research involved designing a service where parents of children with EB would be the main contact with service providers regarding their child's care, it was felt that the parental perspective sufficiently answered the overall research question. However, it would be imperative that future research examine the psychosocial needs of children with EB, especially before implementing and designing services that children themselves would directly access. This research would benefit from the inclusion of children with EB as research participants, as well as informed co‐designers at each step of the research process.

Another limitation of the present study is that a convenience sample was used meaning that participants self‐selected rather than being purposively sampled. This method was selected because it was the most effective and efficient way to answer the overall research question within the resource/time constraints of the study. It may therefore be the case that only those with enough resources (eg time and support) were able to participate, and that others who did not participate may have different understandings of support needed to manage EB. While this may have limited the range of experiences shared, we believe that the saturation observed in the thematic analysis is evidence of an accurate representation among this group.

## 5 CONCLUSION

The purpose of this paper was to provide a report of a study, which was designed to identify the specific health‐care needs of people with EB with a view towards informing the development of appropriate EB‐related health‐care services. A qualitative participatory action research approach guided the design of the study while thematic analysis of the data identified five distinct health‐care care needs. These needs related to (a) support managing physical health‐care issues; (b) access to community/home‐based services; (c) EB‐specific information and psychosocial support; (d) effective interaction with health‐care professionals; and (e) advice regarding benefits and entitlements. The key findings from this study were used to lobby for the allocation of additional resources for individuals and families living with EB. The patient advocacy group, DEBRA Ireland, spearheaded the lobbying process at a national level while the research steering committee was instrumental in designing this study so that it was both appropriate and relevant for people with EB. In this way, a participatory action research approach, while challenging, enabled relatively disparate yet interconnected stakeholders to work together towards achieving a unified aim, that of working towards improving the health and well‐being of people with EB on the island of Ireland.

## CONFLICT OF INTEREST

The authors declare that they have no conflicts of interest.

## AUTHOR CONTRIBUTIONS

EM, AD and SK all contributed to the design of the study. SK collected and analysed the data. SK drafted the paper, and EM, and AD commented and contributed to the revision. All authors read and approved the final version.

## Data Availability

The data collected will not be publicly available or accessible because it could lead to the identification of participants as only a small number of individuals with this condition live in Ireland.
